# Prognostic Impact of Metastasis Timing in Metastatic Gastrointestinal Stromal Tumors: A Multicenter Retrospective Cohort Study

**DOI:** 10.1002/ags3.70245

**Published:** 2026-07-03

**Authors:** Kunihiko Kawai, Tsuyoshi Takahashi, Ryugo Teranishi, Shinsuke Sato, Yukinori Kurokawa, Toshirou Nishida, Seiichi Hirota, Jyunya Fujita, Yuichiro Doki, Toshimasa Tsujinaka

**Affiliations:** ^1^ Department of Gastroenterological Surgery the University of Osaka, Graduate School of Medicine Suita Osaka Japan; ^2^ Department of Gastroenterological Surgery Shizuoka General Hospital Shizuoka City Shizuoka Japan; ^3^ Japan Community Health Care Organization, Osaka Hospital Osaka Japan; ^4^ Department of Pathology Hyogo Medical University Nishinomiya City Hyogo Japan; ^5^ Department of Surgery Yao Municipal Hospital Yao City Osaka Japan; ^6^ Department of Surgery Izumi City General Medical Center Izumi City Osaka Japan

**Keywords:** gastrointestinal stromal tumor, metachronous metastasis, recurrence interval, synchronous metastasis, timing of metastasis

## Abstract

**Background:**

Gastrointestinal stromal tumors (GISTs) commonly metastasize to the liver and peritoneum. Metastases may be detected synchronously at the time of initial diagnosis or develop metachronously after curative‐intent resection. Nonetheless, the prognostic significance of metastasis timing in advanced GIST remains unclear.

**Methods:**

We retrospectively analyzed 147 patients with metastatic GIST treated at multiple institutions between 2003 and 2012. The patients were categorized into synchronous (*n* = 51) and metachronous (*n* = 96) groups. Clinicopathological characteristics and overall survival (OS) were compared using Kaplan–Meier analysis and Cox proportional hazards models. Among patients with metachronous disease, the outcomes were further evaluated according to the recurrence interval.

**Results:**

The median OS was significantly shorter in the synchronous group (6.7 years) than in the metachronous group (12.8 years; *p* = 0.001). Metastasis timing remained an independent prognostic factor in multivariable analysis (hazard ratio 2.36, 95% confidence interval 1.39–4.00; *p* = 0.001). Among patients with metachronous metastasis, early recurrence within 2 years after surgery was associated with significantly inferior survival compared to late recurrence.

**Conclusion:**

Metastasis timing was associated with survival in patients with metastatic GIST. Both synchronous presentation and early metachronous recurrence were associated with poorer outcomes.

## Abstract 2

The clinical course of metastatic gastrointestinal stromal tumors varies according to the timing of metastasis. In this multicenter retrospective cohort study, synchronous metastasis and early metachronous recurrence were associated with poorer survival than late recurrence. Metastasis timing may therefore provide clinically relevant prognostic information in metastatic GIST, although differences in treatment exposure should be considered when interpreting these findings.

## 1 Introduction

Gastrointestinal stromal tumors (GISTs) are the most common mesenchymal neoplasms of the gastrointestinal tract, accounting for approximately 1%–2% of all gastrointestinal malignancies [1]. They are characterized by activating mutations in *KIT* or *PDGFRA* and most commonly arise in the stomach or small intestine [2]. Clinical behavior varies widely, ranging from indolent disease to highly aggressive tumors, depending on tumor size, mitotic activity, anatomical site, and mutation subtype [3, 4, 5].

The introduction of tyrosine kinase inhibitors (TKIs), particularly imatinib, has dramatically improved the outcomes in patients with unresectable or recurrent GIST [6]. Although complete surgical resection remains the standard treatment for localized disease [7], recurrence occurs in a substantial proportion of high‐risk patients. In the metastatic setting, sequential TKIs, such as sunitinib and regorafenib, prolong survival [8]; nonetheless, secondary resistance caused by clonal evolution remains inevitable in most cases [9, 10]. Consequently, metastatic GIST has evolved into a chronic but ultimately progressive disease in the TKI era.

Several large scale registry studies have provided valuable insight into the natural history and treatment patterns of GIST [11, 12]. In Japan, the Kinki GIST Registry, a multicenter observational study conducted across 46 institutions, has collected data from more than 1 200 patients over two registration periods (2003–2007 and 2008–2012) [3, 13]. These data have clarified recurrence risk stratification and real‐world treatment strategies. Nevertheless, most registry analyses have focused on risk classification, mutation status, and overall treatment outcomes, while the prognostic significance of the timing of metastatic disease onset has not been fully examined.

In several advanced GIST studies, patients with metastatic disease at initial presentation and those who develop recurrence following prior curative‐intent resection are often analyzed together as a single “advanced/metastatic” population and treated similarly with TKIs in clinical practice guidelines [14]. However, across multiple solid tumors, the distinction between synchronous and metachronous metastases has been found to reflect the underlying disease biology and influence prognosis and therapeutic strategies. For example, in metastatic renal cell carcinoma, metachronous disease has been associated with more favorable outcomes than synchronous disease in the targeted‐therapy era [15] and remains prognostically relevant in the combination era of immune checkpoint inhibitors [16]. Similar observations have been reported in other malignancies, such as non‐small cell lung cancer with brain metastases [17], and metastatic pancreatic adenocarcinoma [18]. Therefore, metastasis timing can provide clinically relevant prognostic information beyond the conventional staging. In GIST, where long‐term TKI exposure can drive clonal evolution and secondary resistance, synchronous and metachronous metastatic disease may represent distinct clinical entities with different treatment responses and survival outcomes. Nonetheless, this has not been systematically evaluated in large real‐world cohorts.

Therefore, in this study, we aimed to investigate the clinical significance of metastasis timing in patients with advanced GIST using data from a multicenter Japanese registry. Specifically, survival outcomes and treatment responses were compared between patients presenting with synchronous metastatic disease and those who developed metachronous metastases after prior curative‐intent resection. A secondary analysis restricted to patients with metachronous metastases examined whether the interval to recurrence was associated with subsequent treatment outcomes and survival. In our previous analysis of recurrent GIST following curative resection [19], approximately 60% of recurrences occurred within 2 years postoperatively, suggesting a clustering of early relapse events; thus, a 2‐year cutoff was used to explore potential biological and prognostic differences within the metachronous group. By addressing these complementary but distinct questions, we sought to determine whether metastasis timing provides additional prognostic information in the TKI era.

## 2 Methods

### 2.1 Patient Selection and Study Cohort

From 2003 to 2012, 1 426 patients with GIST were registered in the Kinki GIST Study Group database. Of these, 107 patients were excluded because of insufficient clinical or pathological data. Furthermore, 1 172 patients had no metastases.

**FIGURE 1:**
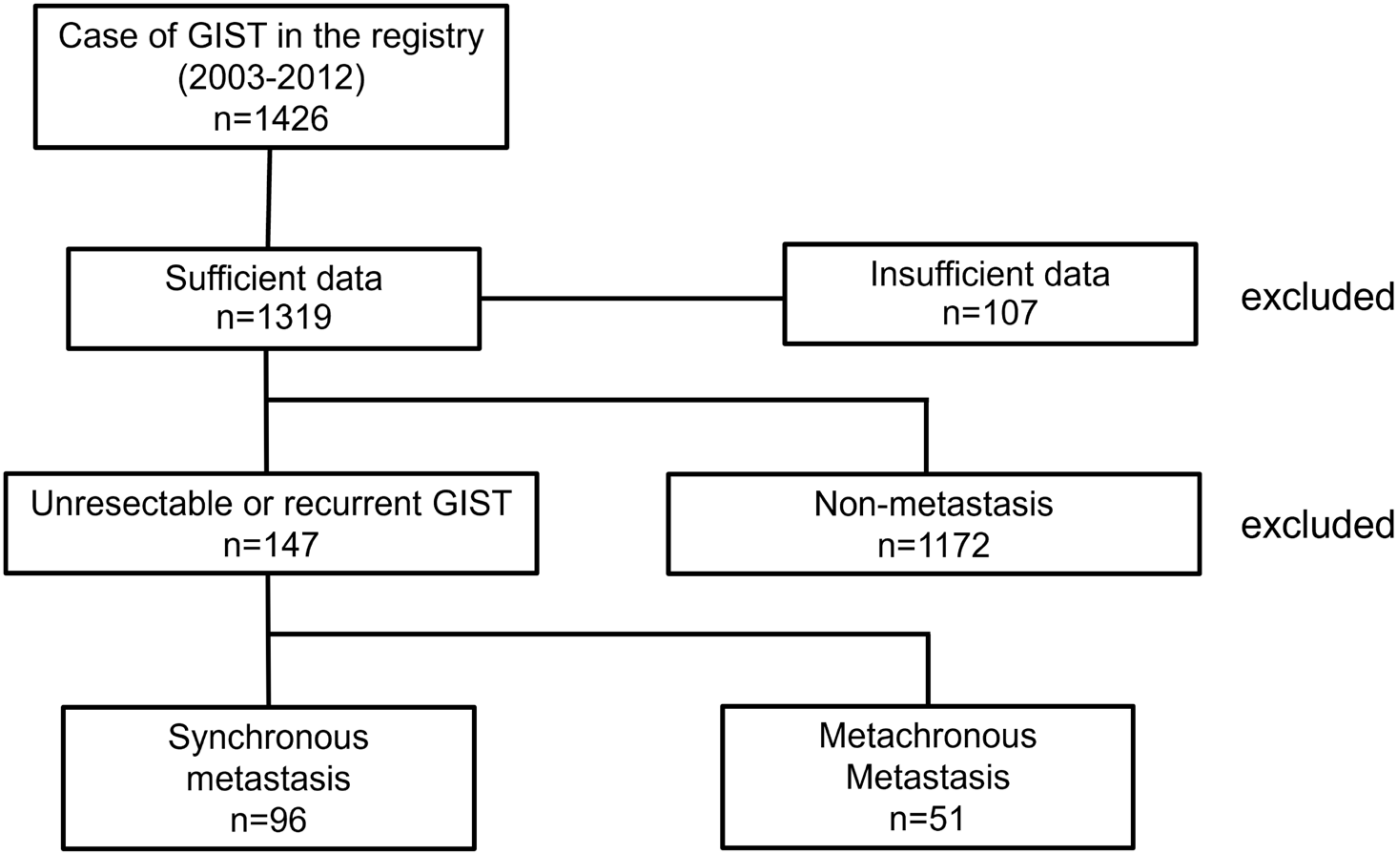
Study recruitment flowchart.

Ultimately, 147 patients with metastatic GIST, who either presented with metastatic disease at initial diagnosis or developed metastases after prior surgical resection, were included in the final analysis. The patient selection process is summarized in Figure 1.

### 2.2 Metastasis Timing Definition

Patients were categorized by metastasis timing. Synchronous metastasis was defined as metastatic disease identified at or before the initial diagnosis of the primary GIST based on the staging workup performed at presentation. Metastatic lesions detected after curative‐intent surgery were classified as metachronous recurrence, regardless of the interval after surgery.

For the subgroup analysis, patients with metachronous metastasis were stratified according to the interval between primary tumor resection and recurrence. Early recurrence was defined as recurrence within 2 years postoperatively, whereas late recurrence was defined as recurrence occurring more than 2 years postoperatively. The 2‐year cutoff was selected based on previous clinical observations [19] and as a clinically practical threshold to distinguish early recurrence after curative‐intent resection.

### 2.3 Data Collection and Variables

Clinical data, including age at metastatic disease diagnosis, sex, primary tumor location, tumor size at initial diagnosis, metastatic sites, and follow‐up duration, were retrospectively collected from participating institutions. Pathological parameters, including the mitotic index (per 50 high‐power fields), were recorded when available. The Ki‐67 labeling index and mutational status (e.g., *KIT* and *PDGFRA*) were not routinely assessed at most participating institutions, particularly during the early years of the registry, and were therefore excluded from the analysis.

Treatment‐related variables included primary tumor resection and adjuvant imatinib administered after curative‐intent surgery. Detailed information on systemic therapy administered after metastatic disease developed was not consistently captured in the registry and was unavailable for analysis.

### 2.4 Statistical Analysis

Data were analyzed using JMP 17 software (SAS Institute Inc., Cary, NC, USA). The primary endpoint was overall survival (OS), defined as the interval from diagnosis of metastatic disease to death from any cause or last follow‐up. OS was compared between patients with synchronous and metachronous metastases. Secondary analyses included subgroup comparisons of OS by sex, age, primary tumor location, and metastatic site, as well as assessment of the prognostic impact of recurrence timing (early vs. late) within the metachronous metastasis group.

Survival curves were estimated using the Kaplan–Meier method and were compared using the log‐rank test. Multivariable analysis was performed using a Cox proportional hazards model to identify independent prognostic factors. Variables entered into the model included age, sex, primary tumor size, mitotic index (when available), and metastasis timing. Hazard ratios (HRs) with 95% confidence intervals (CIs) were calculated. Statistical significance was defined as a two‐sided *p* < 0.05.

### 2.5 Ethical Considerations

The Human Ethics Review Committee of the University of Osaka Hospital approved this study (No. 18424–2), and the Institutional Review Boards of each participating institution approved the study protocol. Because of the retrospective study design, the requirement for written informed consent was waived in accordance with institutional guidelines and national regulations.

## 3 Results

### 3.1 Patients' Characteristics

The patient selection process is illustrated in Figure 1. Overall, 147 patients with metastatic GIST were included in the final analysis, including 51 with synchronous metastasis and 96 with metachronous metastasis (Table 1). Baseline characteristics were broadly comparable between the two groups. The median age was 65 years in both groups, and the proportions of male patients were 51.0% and 59.3% in the synchronous and metachronous groups, respectively. Primary tumor location did not differ substantially between groups. Liver metastasis was observed in 54.9% and 64.6% of synchronous and metachronous cases, respectively, whereas peritoneal metastasis was more common in the synchronous group (54.9% vs. 33.3%). Median primary tumor size was larger in the synchronous than in the metachronous group (12 vs. 7.5 cm). Consistent with this finding, a categorical comparison using a 50‐mm cutoff showed that the synchronous group had a higher proportion of primary tumors ≥ 50 mm than the metachronous group. Mitotic count was similar between groups. Among metachronous cases, the median time to recurrence was 19.5 months, and 26.1% received adjuvant imatinib therapy.

**TABLE 1:** Baseline characteristics according to metastasis timing (synchronous vs. metachronous) in patients with metastatic GIST.

	Synchronous *n* = 51		Metachronous *n* = 96		*p* value
Age, yr^a^					
Median (range)	65	(23–88)	65	(33–82)	0.822
Sex, *n (%)*					
Male	26	(51%)	57	(59%)	0.607
Female	24	(47%)	37	(39%)	
Location of primary lesion, *n (%)*					
Stomach	23	(45%)	48	(50%)	0.218
Small intestine	18	(35%)	39	(41%)	
Others	10	(20%)	9	(9%)	
Location of metastatic lesion, *n (%)*					
Liver	28	(55%)	62	(65%)	
Peritoneum	28	(55%)	32	(33%)	
Others	7	(14%)	19	(20%)	
Time to recurrence					
Median (months)	—	—	19.5	(1–104)	
Tumor size (cm), median (range)	12	(2–35)	7.5	(1–25)	
≧5/< 5	46/5		74/22		0.041
Mitotic count, (/50HPF), median (range)^b^	10	(0–155)	10	(0–160)	
≧5/< 5	35/16		70/13		0.978
Adjuvant therapy					
+/−	—		25 (26%)/71 (74%)		—
Duration of adjuvant therapy (months)					—
Median (range)	—	—	9.0	(0.3–44.1)	

^a^ Data are missing for 15 patients regarding age and for 3 patients regarding sex.

^b^ Data are missing for 14 patients.

Among patients with metachronous metastases (*n* = 96), 55 and 41 were classified as having early recurrence (within 2 years) and late recurrence (after 2 years), respectively (Table 2). Baseline clinicopathologic characteristics were largely similar between the early and late recurrence groups. No substantial differences were observed in age, sex distribution, primary tumor location, metastatic sites, tumor size, mitotic count, or receipt of adjuvant therapy.

**TABLE 2:** Baseline characteristics according to recurrence timing (early vs. late) in patients with metachronous metastatic GIST.

	Early recurrence (*n* = 55)		Late recurrence (*n* = 41)		*p* value
Age, yr^a^					
Median (range)	64	(33–80)	65	(36–82)	0.115
Sex, *n*(%)^a^					
Male	34	(62%)	23	(56%)	0.781
Female	21	(38%)	16	(39%)	
Location of primary lesion, *n (%)*					
Stomach	28	(51%)	20	(49%)	0.978
Duodenum/Jejunum/Ileum	22	(40%)	17	(42%)	
Others	5	(9%)	4	(10%)	
Location of metastatic lesion, *n* (%)^b^					
Liver	35	(64%)	27	(66%)	
Peritoneum	18	(33%)	14	(34%)	
Others	10	(18%)	5	(12%)	
Tumor size (mm), median (range)	8.0	(1–21)	10.0	(2.5–25)	
≧5/< 5	41/14		33/8		0.490
Mitotic count, (/50 HPF), median (range)^c^	13	(2–160)	10	(0–150)	
≧5/< 5	42/5		28/8		0.095
Adjuvant therapy					
+/−	14 (25%)/41 (75%)		11 (27%)/30 (73%)		0.879
Duration of adjuvant therapy (months)					
Median (range)	2.3	(0.3–44.1)	11.6	(5.1–16.0)	< 0.001

^a^ Data are missing for 15 patients regarding age and for 3 patients regarding sex.

^b^ Data are duplicated.

^c^ Data are missing for 14 patients.

### 3.2 Survival Analysis (OS)

**FIGURE 2:**
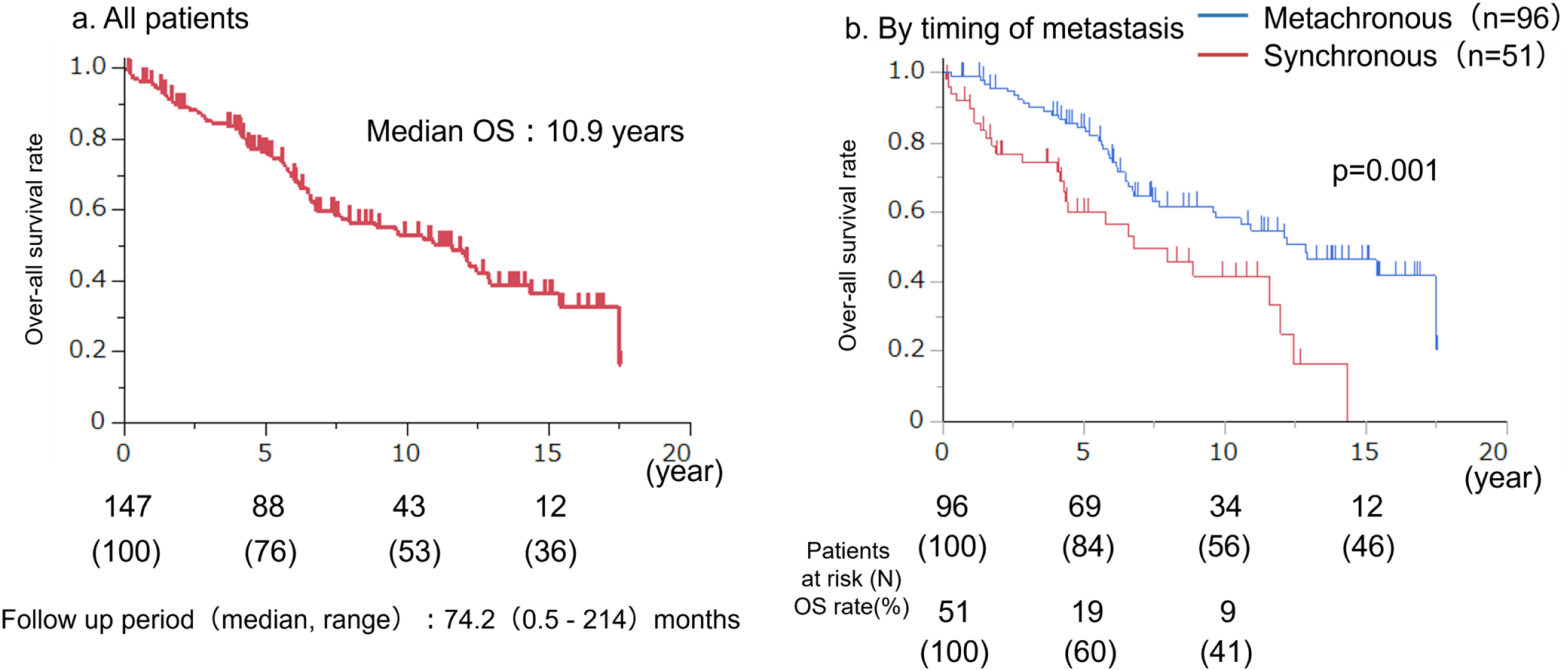
Overall survival of all patients and by metastasis timing. (a) All patients (*N* = 147). (b) By metastasis timing.

OS by metastasis timing is displayed in Figure 2. The median follow‐up duration for the overall cohort was 74.2 months (range, 0.5–214 months). Median OS for the overall cohort was 10.9 years. Patients with metachronous metastasis demonstrated significantly longer survival than did those with synchronous metastasis, with median OS of 12.8 and 6.7 years, respectively (log‐rank *p* = 0.001).

### 3.3 Survival Stratified by Clinically Relevant Subgroups

**FIGURE 3:**
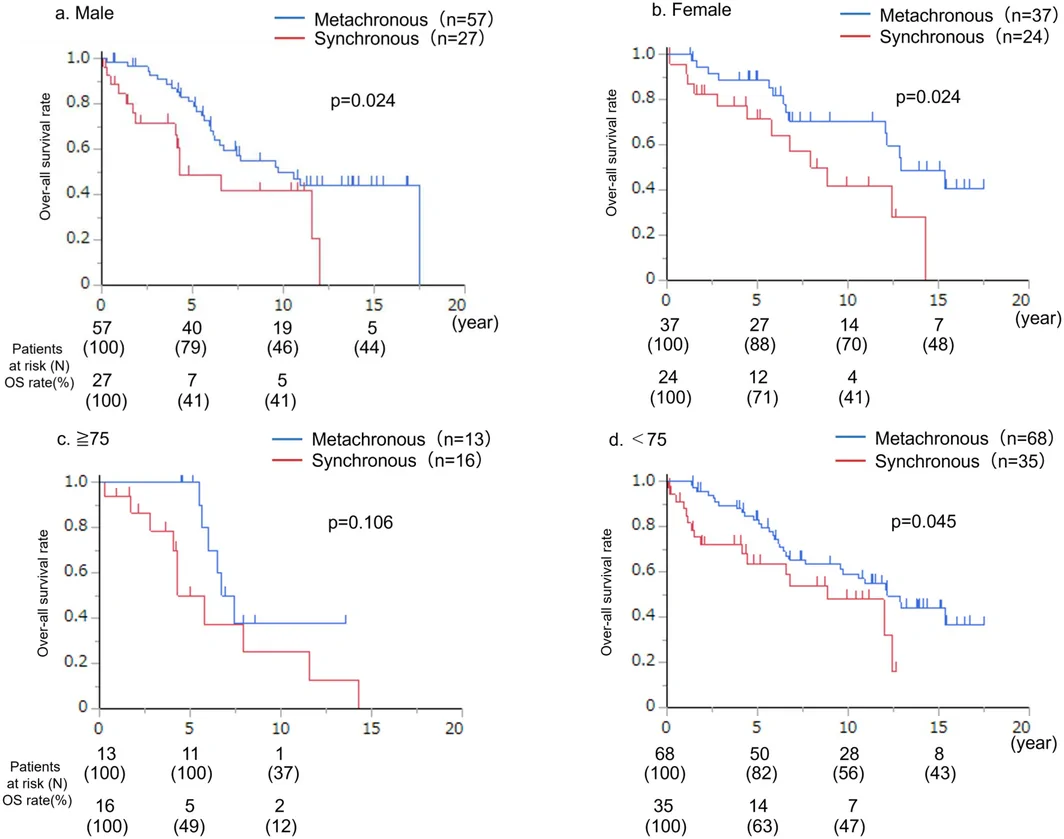
Overall survival by sex and age.

To assess whether the prognostic impact of metastasis timing was consistent across patient subgroups, survival analyses were stratified by sex, age, primary tumor location, and metastatic site. As displayed in Figure 3, the adverse prognostic effect of synchronous metastasis was consistently observed in both male and female patients (*p* = 0.024 for each subgroup).

When stratified by age, the survival difference remained significant in patients aged < 75 years (*p* = 0.045), whereas no statistically significant difference was observed in those aged ≥ 75 years.

**FIGURE 4:**
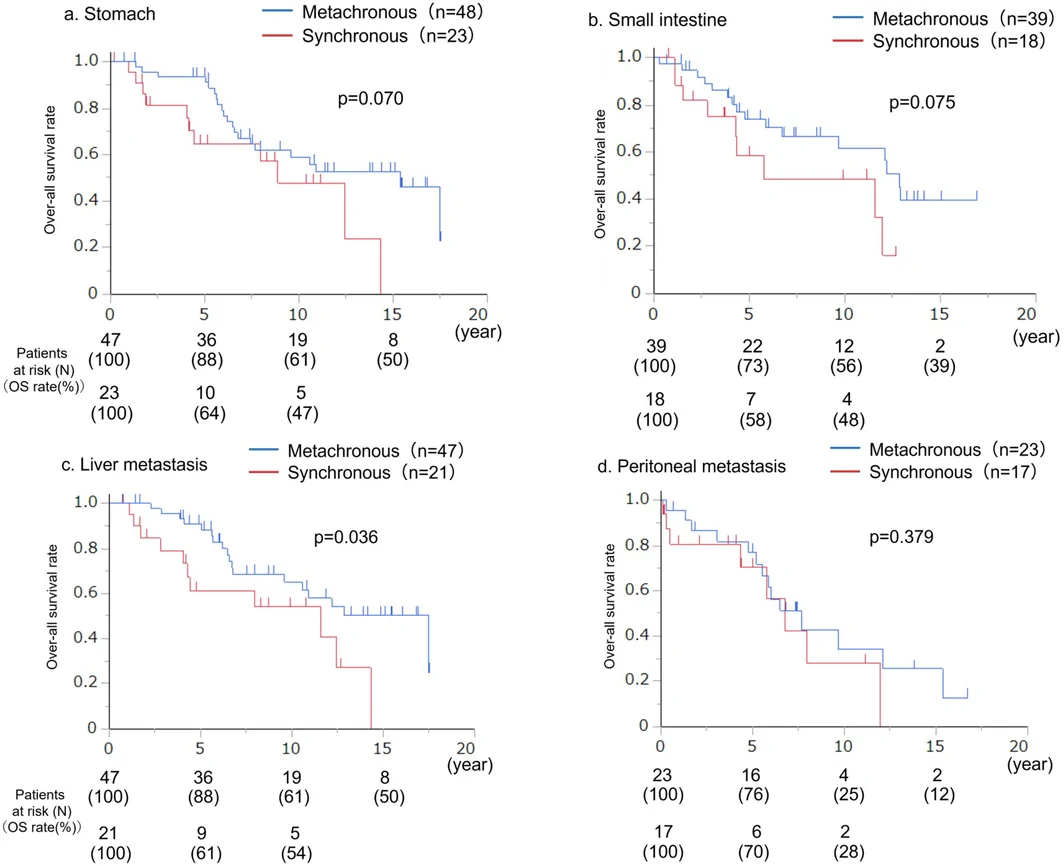
Overall survival by primary site and metastatic site.

Figure 4 shows OS stratified by primary tumor location and metastatic site. In both gastric and small intestinal GIST, metachronous metastasis was associated with a trend toward improved survival compared with synchronous metastasis, although the difference was not statistically significant. Among patients with liver metastases, OS differed significantly between the synchronous and metachronous groups (*p* = 0.036). Conversely, no significant survival difference was observed among patients with peritoneal metastasis.

### 3.4 Multivariate Analysis

In multivariate Cox proportional hazards analysis adjusted for age, primary tumor location, metastatic site, tumor size, and mitotic count, synchronous metastasis remained associated with overall survival (HR 2.36, 95% CI 1.39–4.00, *p* = 0.001) (Table 3). These findings support an association between metastasis timing and survival after adjustment for available clinicopathological factors.

**TABLE 3:** Univariate and multivariate Cox model analysis.

		Univariate analysis		Multivariate analysis	
		HR (95% CI)	*p* value	HR (95% CI)	*p* value
Age, (year)	≥ 75	1.357 (0.731–2.520)	0.332		
Sex	male	1.682 (0.976–2.898)	0.060	1.563 (0.933–2.620)	0.089
Location of primary lesion	small intestine	1.126 (0.646–1.961)	0.674		
Location of metastatic lesion	*p*eritoneum	1.311 (0.772–2.227)	0.315		
Tumor size (mm)	≥ 50	1.183 (0.608–2.304)	0.619		
Mitotic count (/ 50HPF)	≥ 10	1.617 (0.924–2.831)	0.092	1.553 (0.914–2.639)	0.103
Timing of metastasis	synchronous	2.098 (1.208–3.644)	0.008	2.361 (1.393–4.002)	0.001

### 3.5 Prognostic Impact of Recurrence Timing in Metachronous Metastasis

**FIGURE 5:**
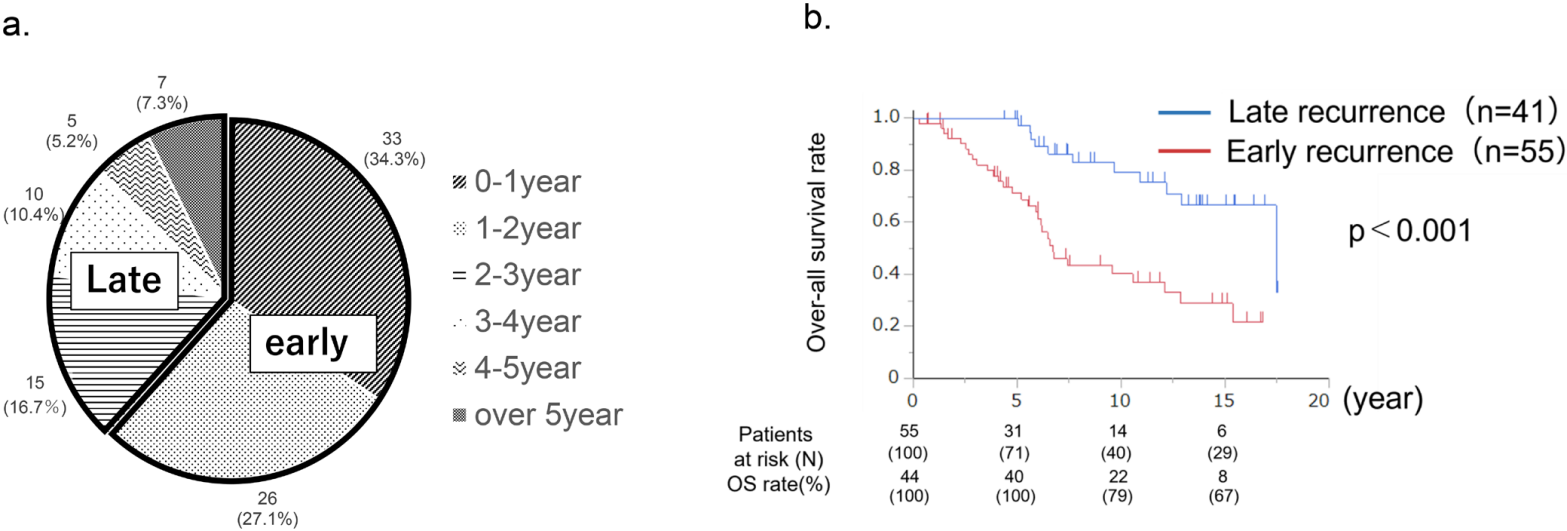
(a) Time to recurrence in cases of metachronous metastasis. (b) Overall survival by recurrence timing. Late: Cases with recurrence more than 2 years after surgery. Early: Cases with recurrence within 2 years after surgery.

Next, whether recurrence timing further stratified the prognosis among patients with metachronous metastasis was examined. The distribution of recurrence timing is presented in Figure 5a. Most recurrences occurred within 2 years postoperatively.

As displayed in Figure 5b, patients with early recurrence (within 2 years postoperatively) demonstrated significantly poorer overall survival compared to those with late recurrence (log‐rank *p* < 0.001). OS did not differ significantly by adjuvant imatinib therapy status within the metachronous group. Mitotic count was also similar between the early and late recurrence groups (*p* = 0.095).

## 4 Discussion

GISTs are biologically heterogeneous neoplasms primarily driven by activating mutations in *KIT* or *PDGFRA* [1, 2, 5]. Although TKIs have substantially improved the prognosis of patients with advanced disease [3, 6], prognostic assessment in metastatic GIST remains largely based on conventional clinicopathological factors [20]. The prognostic relevance of metastasis timing, whether synchronous at initial diagnosis or metachronous after curative‐intent resection, has not yet been systematically assessed. To our knowledge, this study is the first to evaluate the association between metastasis timing and survival in patients with metastatic GIST using a multicenter cohort.

In this multicenter cohort, patients with metachronous metastases exhibited significantly longer overall survival than those with synchronous metastases. Importantly, this association remained significant after adjustment for age, primary tumor location, metastatic site, tumor size, and mitotic count. Thus, metastasis timing may add prognostic information beyond established clinicopathological factors.

Similar observations have been reported in other solid malignancies [15, 16, 17, 18, 21], in which synchronous metastatic presentation has been associated with worse outcomes relative to metachronous disease. Although this study was not designed to directly assess biological mechanisms, the survival differences observed in this cohort suggest that metastasis timing in GIST may reflect clinically meaningful variation in disease course, although this interpretation should be made with caution and may not be fully explained by established clinicopathological factors. When stratified by metastatic site, the survival difference between synchronous and metachronous disease was evident among patients with liver metastasis, but not among those with peritoneal metastasis. This pattern suggests that metastatic site and timing may jointly influence prognosis. A similar site‐dependent pattern has been described in other malignancies [22, 23]. In colorectal cancer, the prognostic impact of metastasis timing appears more pronounced in liver metastasis cohorts, whereas in peritoneal metastasis cohorts, its effect may be attenuated after accounting for disease extent and treatment selection [22]. These findings have been interpreted as reflecting differences in metastatic biology and site‐specific therapeutic feasibility. Although this study was not designed to explore mechanistic explanations, these results align with the possibility that the clinical significance of metastasis timing in GIST may vary by metastatic site. Further investigations incorporating detailed disease burden metrics and molecular profiling are required to clarify this relationship.

Furthermore, metachronous metastasis was not uniformly associated with favorable outcomes. Among patients who developed recurrence after curative‐intent resection, early recurrence within 2 years was strongly associated with poorer OS compared with late recurrence. Similar patterns have been reported in other solid malignancies [23, 24], in which a shorter recurrence‐ or metastasis‐free interval was consistently associated with poorer post‐recurrence survival. Previous studies on GIST have suggested that high mitotic activity is associated with earlier relapse, whereas lower mitotic activity is more often observed in late recurrence [19]. These observations have been interpreted as indicating that early relapse reflects more aggressive tumor biology. However, here, mitotic count did not differ significantly between the early‐ and late‐recurrence groups. This discrepancy suggests that the recurrence interval may capture aspects of disease behavior that are not fully explained by mitotic activity alone. Collectively, these findings support the concept that early relapse represents a clinically important phenotype in GIST. Careful surveillance during the first 2 postoperative years may therefore be particularly relevant, as this interval appears to include a substantial proportion of recurrences. However, the biological validity of this cutoff remains uncertain, and the results should be interpreted with this limitation in mind.

The timing of recurrence in this cohort should also be interpreted in the context of historical practice patterns. During the study period, adjuvant imatinib was either not routinely used or administered for shorter durations than current standards. Conversely, contemporary guidelines recommend at least 3 years of adjuvant therapy for high‐risk GIST [25], and longer durations are being evaluated in selected populations [26]. Because adjuvant imatinib was administered only to patients with metachronous disease following curative‐intent resection, it could not be included in the multivariable model comparing synchronous and metachronous metastasis. In addition, the use and duration of adjuvant therapy were heterogeneous. Therefore, the potential influence of adjuvant imatinib on recurrence timing and subsequent survival cannot be excluded and represents a potential source of bias in this study. Because adjuvant therapy duration was heterogeneous and generally shorter in this cohort, particularly among patients with early recurrence, the potential effect of prolonged adjuvant therapy on recurrence timing and subsequent survival could not be assessed from these data. While its overall impact on survival comparisons between groups is likely limited, this should be interpreted with caution. Whether contemporary extended adjuvant strategies can modify the prognostic implications of early vs. late recurrence remains to be clarified in future studies.

This study had some limitations. First, the retrospective design may have introduced selection bias and unmeasured confounding. Although multivariate analyses were performed to adjust for established clinicopathological factors, residual confounding could not be excluded. Second, the study period should be considered when interpreting the results. Although this study was conducted during a period when TKI therapy, particularly imatinib, had already been introduced and widely adopted as the standard treatment for advanced GIST, treatment strategies were still evolving. Because this was a registry‐based study, detailed information on systemic therapy, including the use, timing, sequence, and duration of TKIs, was not systematically captured. In addition, information on the surgical management of metastatic lesions, including metastasectomy, was not available. Therefore, the impact of treatment heterogeneity on survival outcomes could not be fully assessed, and the influence of treatment differences on the observed survival differences cannot be excluded, representing an important limitation of this study. Third, molecular characteristics, including mutation subtype, were not consistently available across participating institutions. Consequently, whether differences in metastasis timing were associated with specific genomic profiles could not be explored, and their potential prognostic impact could not be fully evaluated. In addition, lesion‐specific data on treatment response and secondary resistance were not available, and their potential contribution to the observed prognostic differences could not be evaluated. Furthermore, because overall survival was defined from the time of metastatic diagnosis, patients with metachronous metastasis must survive until recurrence, which may introduce guarantee‐time bias and potentially favor this group. Finally, although this is a multicenter cohort, certain subgroup analyses were limited by sample size, particularly stratified analyses by metastatic site and age. Therefore, these findings should be interpreted with caution and validated in larger cohorts. Taken together, these limitations underscore the need for cautious interpretation of the findings and limit conclusions regarding the independent biological prognostic significance of metastasis timing.

In conclusion, this multicenter study demonstrated an association between metastasis timing and overall survival in patients with metastatic GIST. Patients with synchronous metastasis showed poorer outcomes than those with metachronous metastasis, and early recurrence within 2 years after resection with curative intent among patients with metachronous disease was associated with inferior survival. However, these findings should be interpreted with caution and considered hypothesis‐generating.

## Author Contributions

**Kunihiko Kawai:** writing – review and editing, writing – original draft, methodology, data curation, investigation, formal analysis, conceptualization, visualization. **Shinsuke Sato:** data curation, resources, writing – review and editing, investigation. **Seiichi Hirota:** writing – review and editing, supervision, conceptualization. **Yukinori Kurokawa:** writing – review and editing, supervision, conceptualization. **Tsuyoshi Takahashi:** conceptualization, methodology, data curation, supervision, formal analysis, investigation, writing – original draft, writing – review and editing, project administration. **Toshimasa Tsujinaka:** conceptualization, supervision, writing – review and editing. **Ryugo Teranishi:** investigation, resources, data curation, writing – review and editing. **Jyunya Fujita:** conceptualization, supervision, writing – review and editing. **Toshirou Nishida:** supervision, writing – review and editing, conceptualization. **Yuichiro Doki:** conceptualization, supervision, writing – review and editing.

## Funding

The authors have nothing to report.

## Ethics Statement

The Human Ethics Review Committee of the University of Osaka Hospital approved this study (No. 18424–2), and the institutional review board of each participating institution approved the study protocol.

## Conflicts of Interest

The authors have no conflicts of interest or financial ties to any of the firms mentioned in this report. Y. Kurokawa is an Associate Editor of Annals of Gastroenterological Surgery.

## Data Availability

The data that support the findings of this study are available on request from the corresponding author. The data are not publicly available due to privacy or ethical restrictions.
